# Immediate frozen-embryo transfer: a viable option after hysteroscopic polypectomy to shorten time to pregnancy without compromising live birth rate

**DOI:** 10.3389/frph.2026.1754874

**Published:** 2026-01-27

**Authors:** Hongxiang Sun, Deying Ban, Chen Wang, Hui Chen

**Affiliations:** 1 Clinical Research Center for Reproduction and Genetics in Hunan Province, Reproductive & Genetic Hospital of CITIC-Xiangya, Changsha, China; 2 NHC Key Laboratory of Human Stem Cell and Reproductive Engineering, School of Basic Medical Sciences, Central South University, Changsha, China

**Keywords:** endometrial polyp, frozen-thawed embryo transfer, hysteroscopy, pregnancy outcomes, timing of transplantation

## Abstract

**Objective:**

To compare the effects of frozen-thawed embryo transfer at different times after hysteroscopic endometrial polyp resection on pregnancy outcomes and to provide evidence for individualized clinical treatment.

**Methods:**

This study was a single-center retrospective cohort study involving 756 infertile patients who underwent hysteroscopic polypectomy for endometrial polyps from 2023 to 2024 and underwent natural cycle frozen-thawed embryo transfer for the first time after surgery, divided into two groups: Group A (178 cases) underwent transfer during the menstrual cycle after the operation, while Group B (578 cases) underwent transfer after next menses after the operation. Baseline characteristics, clinical pregnancy rate, live birth rate and miscarriage rate were compared between the two groups. Additionally, a correlation analysis between the interval from surgery to embryo transfer and pregnancy outcomes in the immediate transfer group.

**Results:**

There were no significant differences in baseline characteristics such as age, BMI, AMH level between the two groups. Unadjusted, there were no statistically significant differences in clinical pregnancy rate (60.7% vs. 58.3%), live birth rate (57.9% vs. 56.4%), early miscarriage rate (2.8% vs. 8.9%), and late miscarriage rate (1.9% vs. 2.7%) between group A and group B (all *P* > 0.05). After adjusting for confounding factors such as age, prevalence of endometritis, and type of transplanted embryo, the timing of transplantation still had no significant effect on pregnancy outcomes (clinical pregnancy rate OR = 0.99, 95% CI: 0.68–1.42; live birth rate OR = 1.02, 95% CI: 0.71–1.46). However, binary logistic regression showed a negative correlation between maternal age and pregnancy rate and live birth rate. In group A, the interval from surgery to transplantation was not significantly correlated with clinical pregnancy and live birth outcome.

**Conclusion:**

There was no significant difference in pregnancy outcomes between frozen-thawed embryo transfer in the current menstrual cycle and the next menstrual cycle after hysteroscopic endometrial polyp resection, and the choice of transfer timing was not a key factor affecting pregnancy outcomes. For patients with special clinical needs, transplanting in the same menstrual cycle after surgery is a feasible option, which helps to shorten the waiting time and reduce mental stress.

## 1 Introduction

Endometrial polyps are a common disease among women of childbearing age, with a significantly higher prevalence of up to 8% among infertile women (1). As one of the important factors causing infertility, endometrial polyps may interfere with embryo implantation through various mechanisms such as mechanical obstruction, triggering local inflammatory responses, and altering endometrial receptivity, thereby negatively affecting the outcome of assisted reproductive technology (ART) (2). Hysteroscopic resection of endometrial polyps is currently the gold standard for the diagnosis and treatment of endometrial polyps, which can effectively improve the uterine cavity environment and increase the fertility potential of patients.

For patients who need *in vitro* fertilization-embryo transfer assistance, when to perform frozen-thawed embryo transfer after polypectomy for the best pregnancy outcome is one of the key issues in clinical decision-making. Theoretically, performing embryo transfer during the menstrual cycle after surgery can take full advantage of the window of improvement in the endometrium after polyp removal, shorten the waiting time for treatment, and reduce the psychological burden on patients. This option is particularly suitable for two common clinical scenarios: one is patients whose polyps are detected during fertility cycle monitoring and who do not wish to cancel the cycle after surgery and do not want to wait for the next menstrual cycle; Patients who seek medical treatment from a distance and wish to complete the surgery and transplant in one go to save costs. On the other hand, however, endometrial polyp removal itself may cause short-term trauma and inflammatory responses to the endometrium, requiring a certain repair process. Therefore, there is a view that delaying a menstrual cycle may provide more time for endometrial repair and thus be more conducive to embryo implantation.

Studies showed that fertilization rates, implantation rates, and clinical pregnancy rates were similar in both groups (3) that received *in vitro* fertilization (IVF) less than 6 months after polyp removal and those that received IVF more than 6 months after polyp removal. Frozen-thawed embryo transfer (FET) in the first menstrual cycle after hysteroscopic resection of endometrial polyps showed no significant difference in pregnancy outcomes compared with transplanting at longer intervals, such as 2–3 menstrual cycles (4). At present, there is limited direct comparison evidence between FET performed in the current menstrual cycle and in the next menstrual cycle after endometrial polyps (EPs) resection.

Therefore, this study aims to compare the impact of initiating FET in the current menstrual cycle and the next menstrual cycle after hysteroscopic endometrial polyp resection on pregnancy outcomes through a retrospective cohort analysis, with a focus on evaluating indicators such as clinical pregnancy rate, live birth rate, and miscarriage rate, in order to provide evidence-based evidence for the timing of transplantation under specific clinical needs and optimize individualized treatment strategies.

## 2 Materials and methods

### 2.1 Research design and subjects

The research protocol was approved by the Institutional Review Board of Reproductive & Genetic Hospital of CITIC-Xiangya. The study was a single-center retrospective cohort study that retrospectively analyzed the clinical data of infertile patients who underwent hysteroscopic resection for EPs at Reproductive & Genetic Hospital of CITIC-Xiangya from 2023 to 2024 and underwent first natural cycle FET after the procedure.

Inclusion criteria: (1) Assisted reproductive technology for infertility, using a total embryo cryopreservation strategy; (2) Transfer using the natural cycle endometrial preparation protocol; (3) Endometrial polyps are diagnosed by transvaginal ultrasound and hysteroscopic polypectomy is performed before FET, and the postoperative pathology confirms endometrial polyps; (4) The first FET after the operation was a transfer of high-quality cleavage-stage embryos or blastocysts.

Exclusion criteria: (1) Other surgeries were performed concurrently during the operation, such as surgeries for intrauterine adhesions, hydrosalpinx, or uterine fibroids; (2) Presence of untreated hydrosalpinx; (3) Lost to follow-up pregnancy outcomes.

### 2.2 Study grouping

Patients were divided into two groups based on the timing of transplantation after hysteroscopic polypectomy: Group A: entered the FET cycle in the same menstrual cycle after hysteroscopic polypectomy. Group B: Entered the FET cycle after the first menstrual period following hysteroscopic polypectomy.

### 2.3 Hysteroscopic endometrial polypectomy

All hysteroscopic procedures were performed by senior physicians under intravenous general anesthesia between 3 and 7 days after the cessation of menstruation. Depending on EPs characteristics (such as size and number) and patient conditions (medical history and personal preference), surgeries were conducted either in the outpatient surgical center or inpatient operating room. A standardized protocol was followed: initial assessment of the uterine cavity was performed using a diagnostic hysteroscope (Karl Storz, Germany). Upon identification of polyps, gentle mechanical cervical dilation was uniformly applied until the hysteroscope sheath could be passed smoothly, followed by conversion to operative hysteroscopy. Using a rigid hysteroscope (outer sheath diameter approximately 6.5 mm) with normal saline as the distension medium, polyps were completely resected at their base under direct visualization using cold scissors or grasping forceps to avoid electrothermal damage to the surrounding normal endometrium. Multiple polyps were defined as the presence of two or more polyps. All specimens were fixed in formalin and sent for pathological examination.

### 2.4 Embryo transfer

All embryos were transferred after frozen-thawing using the natural cycle endometrial preparation protocol. 1–2 embryos are transferred per cycle, and the transfer procedures are performed by a fixed team of physicians. For Group A, the operation day was taken as the starting monitoring point for their natural cycle FET, and the embryo transfer day was subsequently determined based on follicular development and the condition of the endometrium.

### 2.5 Pregnancy monitoring

Serum hCG levels are measured 12 or 14 days after embryo transfer, and the gestational sac is detected by transvaginal ultrasound 26 or 28 days after transfer. And continue to monitor and follow up during pregnancy until the end of the patient's pregnancy. Clinical pregnancy is defined as a gestational sac with original heartbeats visible on B-ultrasound 26 or 28 days after transplantation. Live birth is defined as a viable delivery after 28 weeks of gestation. Abortion is defined as any loss of pregnancy after clinical pregnancy. Early abortion is defined as an abortion that occurs before 12 weeks of gestation, and late abortion is defined as an abortion that occurs between 12 weeks and less than 28 weeks of gestation.

### 2.6 Observation indicators

Baseline data: Age, body mass index (BMI), waist-to-hip ratio, AMH, whether combined with polycystic ovary syndrome/insulin resistance/abnormal glucose metabolism/adenomyosis/endometritis, and the number of polyps were collected in both groups of patients.

Embryo transfer indicators: The timing of transfer, the interval from surgery to transplantation, the type and number of embryos transferred, and the endometrial thickness on the day of transfer were collected for both groups of patients.

Outcome indicators: Live birth rate, clinical pregnancy rate, early miscarriage rate, late miscarriage rate. Clinical pregnancy rate = (number of clinical pregnancy cycles/total number of transfer cycles) × 100%. Live birth rate = (number of live delivery cycles/total number of transfer cycles) × 100%. Early abortion rate = (number of early abortion cycles/number of clinical pregnancy cycles) × 100%. Late miscarriage rate = (number of late miscarriage cycles/clinical pregnancy cycles) × 100%.

### 2.7 Statistical analysis

Data analysis was performed using SPSS 26.0 software. Measurement data were expressed as mean ± standard deviation if they were normally distributed, and the independent sample *t*-test was used for comparison between groups; Continuous variables with a non-normal distribution were presented as median (interquartile range) and compared using the Mann–Whitney *U* test. Count data were expressed as rates (%), and the *χ*² test was used for comparison between groups. To control for the effect of baseline confounding factors, binary Logistic regression was used to correct for baseline indicators with differences between groups. Furthermore, for the immediate transfer group, Spearman rank correlation test was used to evaluate the association between the interval from surgery to embryo transfer and both clinical pregnancy and live birth outcomes. A two-sided *P*-value <0.05 was considered statistically significant.

### 2.8 Ethical approval

This study is a retrospective observational study and has been approved by the Reproductive & Genetic Hospital of CITIC-Xiangya (study code: LL-SC-2019-023). All research procedures comply with the ethical principles of the Declaration of Helsinki. As the study only involves anonymous analysis of existing clinical data and does not pose any new risks or interventions to patients, the ethics committee has approved the exemption from obtaining individual informed consent from patients.

## 3 Results

### 3.1 Comparison of baseline characteristics of patients

**Figure 1:**
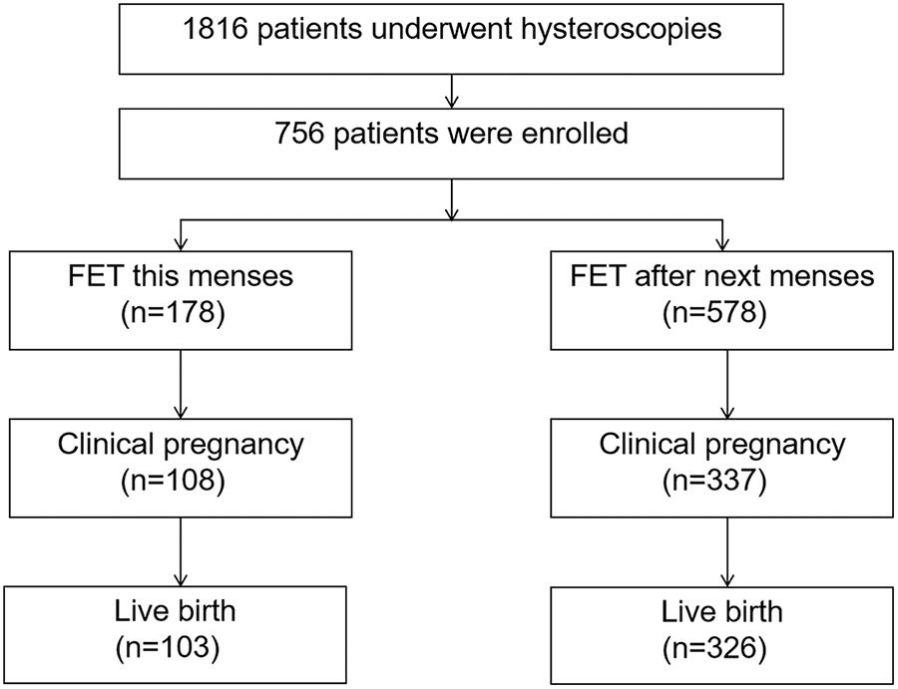
Study flowchart.

A total of 1,816 infertile patients underwent hysteroscopic endometrial polyposectomy during the study period. The 756 patients who met the inclusion criteria were divided into two groups: Group A with 178 cases, with a median interval from hysteroscopic surgery to embryo transfer of 13 days (range: 8–20 days) and Group B with 578 cases, with a median interval of 39 days, which was significantly longer than that in Group A (*P* < 0.001). Figure 1 shows the flowchart of the study.

There were no statistically significant differences (*P* > 0.05) between the two groups of patients in terms of age, BMI, waist-hip ratio, AMH level, whether combined with polycystic ovary syndrome/insulin resistance/abnormal glucose metabolism/adenomyosis, the number of polyps, the number of transplanted embryos, and the baseline data of endometrial thickness on the day of transplantation. The proportion of patients with endometritis in group A was significantly lower than that in group B (19.1% vs. 50.3%, *P* = 0.00), while the proportion of blastocysts transplanted in group A was higher than that in group B (41.6% vs. 23.5%, *P* = 0.00). These two factors were differential factors between the groups and will be corrected in the subsequent analysis. See Table 1.

**Table 1:** Patient baseline characteristics and embryo transfer details.

Variable	FET this menses (*n* = 178)	FET after next menses (*n* = 578)	*P*
Age (years)	32.46 ± 4.43	33.09 ± 4.29	0.09
BMI (kg/m^2^)	21.90 ± 2.51	22.01 ± 2.56	0.62
Waist-to-hip ratio	0.81 ± 0.06	0.80 ± 0.06	0.27
AMH (ng/mL)	3.93 ± 2.76	3.95 ± 3.10	0.95
Coexisting PCOS	12 (6.7%)	29 (5.0%)	0.37
Coexisting insulin resistance	100 (56.2%)	331 (57.3%)	0.80
Coexisting glucose metabolism disorder	17 (9.6%)	73 (12.6%)	0.27
Coexisting adenomyosis	31 (17.4%)	110 (19.0%)	0.63
Coexisting endometritis	34 (19.1%)	291 (50.3%)	0.00
Number of EPs			0.11
Single	23	51	
Multiple	155	527	
Number of embryos transferred			0.06
1	136	400	
2	42	178	
Embryo type			0.00
Cleavage-stage embryo	104	442	
Blastocyst	74	136	
Endometrial thickness on transfer day (mm)	12.38 ± 1.86	12.22 ± 1.72	0.31
Interval from surgery to embryo transfer (days)	13 (11–16)	39 (36–43)	0.00

### 3.2 Comparison of pregnancy outcomes

Unadjusted pregnancy outcomes were compared between the two groups as shown in Table 2: (1) Clinical pregnancy rate: 60.7% (108/178) in group A and 58.3% (337/578) in group B, with no statistically significant difference between the two groups (*P* > 0.05). (2) Live birth rate: 57.9% (103/178) in group A and 56.4% (326/578) in group B, with no statistically significant difference between the two groups (*P* > 0.05). (3) Early abortion rate: 2.8% (3/108) in group A and 8.9% (3/337) in group B, with no statistically significant difference between the two groups (*P* > 0.05). In Group A, all 3 cases of early miscarriage were due to personal reasons of the patients, while in group B, there were 3 cases of early miscarriage, one of which was due to personal reasons. (4) Late miscarriage rate: 1.9% (2/108) in group A and 2.7% (9/337) in group B, with no statistically significant difference between the two groups (*P* > 0.05).

**Table 2:** Comparison of pregnancy outcomes.

Variable	FET this menses (*n* = 178)	FET after next menses (*n* = 578)	*χ* ^2^	*P*
Clinical pregnancy rate	60.7% (108/178)	58.3% (337/578)	0.32	0.57
Early miscarriage rate	2.8% (3/108)	8.9% (3/337)	2.19	0.14
Late miscarriage rate	1.9% (2/108)	2.7% (9/337)	0.23	0.63
Live birth rate	57.9% (103/178)	56.4% (326//578)	0.12	0.73

### 3.3 Comparison of outcomes after adjusting for confounding factors

Table 3 presents the results of binary logistic regression analysis of factors related to pregnancy and live birth. To further clarify the independent impact of transplantation timing on pregnancyand live birth outcomes, factors with *P* values greater than 0.1 between groups in Table 1 were included. “Age, combined endometritis, type of transplanted embryo” and the timing of transplantation were included as variables in the binary Logistic regression model for correction.

**Table 3:** Multivariate logistic regression analysis of pregnancy and live birth outcomes.

Variable	Pregnancy			Live birth		
	OR	(95% CI)	*P*	OR	(95% CI)	*P*
Age	0.15	(0.05–0.47)	0.00	0.12	(0.04–0.38)	0.00
Transfer timing	0.99	(0.68–1.42)	0.94	1.02	(0.71–1.46)	0.93
Coexisting endometritis	0.91	(0.67–1.24)	0.55	0.95	(0.70–1.29)	0.73
Embryo type	1.15	(0.81–1.64)	0.44	1.15	(0.81–1.64)	0.45
Number of embryos transferred	1.10	(0.78–1.55)	0.60	1.11	(0.79–1.56)	0.56

The adjusted analysis showed that the timing of transfer had no statistically significant effect on pregnancy and live birth outcomes (OR = 0.99, 95% CI: 0.68–1.42, *P* > 0.05; OR = 1.02, 95% CI: 0.71–1.46, *P* > 0.05). This suggests that, after balancing the baseline differences, there were no significant differences in clinical pregnancy rate and live birth rate between transplantation in the current menstrual cycle and transplantation in the next menstrual cycle after hysteroscopic endometrial polyp resection. However, it was found that maternal age had a negative impact on pregnancy rate and live birth rate (OR = 0.99, 95% CI: 0.68–1.42, *P* > 0.05; OR = 1.02, 95% CI: 0.71–1.46, *P* > 0.05).

### 3.4 Correlation analysis between the surgery-to-transfer interval and pregnancy outcomes in the immediate transfer group

To further investigate the influence of transfer timing on outcomes in group A, we conducted a correlation analysis between the interval from surgery to embryo transfer and pregnancy outcomes. Spearman rank correlation analysis revealed no significant association between the interval length and either clinical pregnancy outcomes or live birth outcomes, as detailed in Table 4. These findings indicate that, within the postoperative window of 8–20 days, the specific timing of embryo transfer does not affect the ultimate pregnancy success rate. Therefore, the embryo transfer date can be flexibly determined based on individual follicular development and endometrial conditions.

**Table 4:** Spearman correlation analysis between the interval from surgery to embryo transfer and pregnancy outcomes in the immediate transfer group (*n* = 178).

Outcome	*r_s_*	*P*
Clinical pregnancy	0.09	0.24
Live birth	0.08	0.30

## 4 Discussion

This study, through a single-center retrospective cohort analysis, explored the impact of frozen-thawed embryo transfer at different times after hysteroscopic endometrial polyposectomy on pregnancy outcomes. The findings revealed no statistically significant differences in clinical pregnancy rates, live birth rates, or miscarriage rates between patients undergoing FET in the same menstrual cycle after surgery and those undergoing FET after the first postoperative menstruation. This suggests that the timing of embryo transfer does not exert a significant independent effect on live birth outcomes. This study confirmed that performing frozen-thawed embryo transfer within the broad postoperative window of 8–20 days (median: 13 days) after hysteroscopic polypectomy resulted in pregnancy outcomes comparable to those achieved with delayed transfer in the subsequent menstrual cycle, thereby providing clear and clinically feasible evidence to support the application of immediate postoperative cycle transfer strategies.

This main finding is in line with the conclusions of existing studies. A retrospective study showed no significant differences (4) in implantation rate, clinical pregnancy rate, miscarriage rate, and live birth rate among the groups when FET was performed in the first menstrual cycle, two to three menstrual cycles, or more than three menstrual cycles after hysteroscopic polypectomy. This suggests that endometrial repair may quickly reach a good receptive state after hysteroscopy, and the findings of this study further reinforce this evidence and provide more direct evidence for patients with special clinical needs, such as the desire to shorten the treatment cycle and reduce waiting time. Zhu et al. showed that simultaneous embryo transfer and hysteroscopy in the same menstrual cycle did not compromise pregnancy outcomes (5). The study corrected for baseline differences through regression analysis and showed that the timing of embryo transfer had no significant impact on pregnancy outcomes.

Surgical removal of polyps themselves is crucial for improving reproductive outcomes. Polyps may alter endometrial receptivity, affect embryo implantation, and reduce pregnancy rates. Hysteroscopic polyp removal is feasible and safe (6, 7). Studies confirmed that after polyp removal, the pregnancy rate of patients increased while the rate of early miscarriage decreased (2). Therefore, for patients with endometrial polyps who have infertility, hysteroscopic surgery is recognized as the preferred treatment option. However, it should be emphasized that the recurrence rate of endometrial polyps is very high, which has a negative impact on the conception rate. The postoperative recurrence rate of endometrial polyps is between 2.5% and 43.6%. A multivariate logistic regression analysis involving 168 premenopausal women with endometrial polyps who underwent hysteroscopic polypectomy showed that more endometrial polyps and longer follow-up time were significantly associated with an increased risk of postoperative polyp recurrence (8).

The possible reason for the feasibility of immediate transplantation is that hysteroscopic surgery, while removing polyps, may trigger beneficial endometrial repair and regeneration processes through the “endometrial scraping” effect. This minor traumatic response may facilitate the secretion of cytokines and the improvement of endometrial receptivity, thereby creating a microenvironment conducive to embryo implantation and this effect may persist into the subsequent cycle (9, 10). The underlying biological rationale for the feasibility of immediate post-operative transfer may be associated with the rapid endometrial repair and regenerative capacity following intrauterine procedures. This is supported by the study by Pecorino et al. (11), which found that in patients with recurrent implantation failure, endometrial scratching performed in the cycle prior to transfer showed no significant difference in implantation or clinical pregnancy rates compared to a sham procedure group. This result indicates that a single, standardized, and gentle intrauterine procedure does not cause sustained negative effects on endometrial receptivity. This indirectly corroborates the conclusion of the present study: if the surgical procedure is precise and minimally invasive, the endometrium is likely capable of completing repair and achieving a favorable receptive state within a short period after surgery, thereby making immediate transfer a viable option.

In addition, endometrial polyps themselves are a focal lesion, and the improvement of the uterine cavity environment after their removal is direct and rapid. As long as the operation is precise and does not cause excessive damage to the normal endometrial tissue, the function of the endometrium can usually be effectively restored after a period of recovery. However, a study showed that no pregnancies were observed in patients whose interval between hysteroscopic polyp resection and embryo transfer was less than 5 days. Multivariate logistic regression analysis revealed that the polyp resection and the interval between embryo transfer had a significant predictive effect on the live birth rate (12). This suggests that the transplantation should be postponed until the wound has fully healed. In this study, the surgery-transfer interval was ≥8 days for the transfer group in the current cycle, indicating that waiting for the initial repair of the wound before transfer within a reasonable time window does not affect the success rate of pregnancy. Furthermore, the results of this study have clear clinical guiding significance. For patients who are eager to complete the transplantation as soon as possible and shorten the assisted pregnancy time (such as those with advanced age, long-distance medical treatment, or mental anxiety), if the interval between the surgery and the transplantation is reasonable, undergoing FET during the postoperative menstrual cycle is a reasonable and effective choice. This helps to alleviate the mental stress and economic burden of patients during the waiting period. On the other hand, the research results also suggest that if patients need to wait for some time due to personal reasons or their physical conditions (for example, needing time to adjust the endometrium or dealing with other complications after the surgery), delaying the transplantation by one cycle will not have an adverse effect on the final pregnancy success rate. This provides greater flexibility for clinicians to formulate individualized treatment plans.

This study has several limitations. First, the retrospective design may introduce selection bias and residual confounding that cannot be fully controlled. Although we performed statistical adjustments for known confounders, unmeasured factors may still influence the accuracy of the results. Second, as a single-center study, the sample size—particularly in the immediate transfer group—is relatively limited. Moreover, there is a notable imbalance in sample sizes between the two groups, which primarily stems from the inherent constraints of retrospective design and the clinical preference of most patients for delayed transfer. This imbalance may affect the statistical power to some extent. To mitigate its potential impact, we employed the following analytical strategies: first, the two groups were comparable in key baseline characteristics such as age and BMI; second, variables that differed between groups (e.g., the prevalence of endometritis and the proportion of blastocyst transfers) were included in the logistic regression model to adjust for their effects and to assess the independent impact of transfer timing on pregnancy outcomes. Despite these measures, future prospective studies with matched sample sizes are warranted to further validate the findings. Additionally, integrating molecular biology techniques to dynamically monitor the expression of endometrial receptivity markers at different time points after hysteroscopic surgery could help elucidate the mechanisms underlying optimal timing for embryo transfer.

## 5 Conclusion

This study shows that for patients who underwent hysteroscopic polypectomy due to endometrial polyps combined with infertility, the clinical pregnancy rate and live birth rate were comparable when the first frozen-thawed embryo transfer was performed in the current menstrual cycle and the next menstrual cycleafter surgery. This suggests that the timing of the transfer should be based on the individual circumstances and clinical needs of the patient, and there is no need to deliberately delay the transfer while ensuring good healing of the surgical wound. This study provides evidence-based medical evidence for clinicians to offer more flexible and timely individualized fertility assistance strategies to patients under specific conditions.

## Data Availability

The raw data supporting the conclusions of this article will be made available by the authors, without undue reservation.
